# Four Practical Aspects of the Mental Deficiency Act, 1913

**Published:** 1913-10-11

**Authors:** Horatio Bryan Donkin


### FOUR PRACTICAL ASPECTS OF THE MENTAL DEFICIENCY ACT, 1913.

### The Treatment of Defectives and Further Research.

By Sir HORATIO BRYAN DONKIN, M.D., F.R.C.P.

This measure, recently enacted after much unintelligent and obstructive opposition, owes its origin to strong representations made to the Government in power in 1904 by certain public associations whose wide practical experience had fully convinced them of the existence of great evils resulting from the inadequate care and control of large numbers of mentally deficient persons of all ages. Prominent among these associations were the Charity Organisation Society and the National Association for the Care of the Feeble-Minded. A conference of members of several Government Departments and other public officials was then appointed by the Home Office to consider these representations, and to report on the advisability or otherwise of taking steps in the direction indicated. At this conference were represented, among other bodies, the Lunacy Commission, the Prison Commission, the Education Office, and the Local Government Board. After due deliberation the conference reported unanimously to the effect that a *primâ-facie* case for a serious inquiry was established by the evidence before them as well as by their own practical knowledge, and recommended the appointment of a Royal Commission as the most appropriate means of thoroughly investigating the important questions, both administrative and financial, which this subject involved.

### "The Most Important Question."

Having been a member of the preliminary conference (as a representative of the Prison Commission) and also of the Royal Commission which was appointed later in the same year (1904), and having also intimate knowledge, from my medical experience as Prison Commissioner during several years, of the evils caused by the unconditional discharge from prison of numerous mentally defective criminals who could not be certified insane under the existing laws, I shall touch upon this matter first among the remarks that follow concerning the *practical* aspects of the Mental Deficiency Act. But, though I emphasise this aspect first in order, owing to my personal knowledge and active interest in it, I would not be understood to regard it as first in importance. There is certainly one other practical aspect of this Act—viz., that concerning the care and control of incurably "defective" children, which touches, perhaps, the most important question of all: the prevention of much harm both suffered and done by defective persons who from their earliest childhood have been neglected and uncontrolled.

### I. *The Act in its Relation to the Treatment of Mentally Defective Criminals.*

Since this Act provides for the legal recognition and certification of mentally defective persons who, although demonstrably defective, have hitherto been excluded from certification, it will now be possible to treat, rationally and effectively, a class of convicted criminals, amounting probably to upwards of 10 per cent. of the whole, by transferring them to institutions expressly adapted for the care and control of mentally defective persons, instead of retaining them, as at present, in prison until they have either earned their licence or served their sentence, and then giving them their freedom, which they usually forfeit in a short time by the commission of fresh crimes. Ample safeguards are provided by the Act for the due certification of such persons and for the necessary renewal, or cancelment, of such certificates. Thus, the treatment of such criminals will be such as is proper to mentally defective persons; not a penal *régime*, except in so far as a deprivation of liberty is inevitably penal in some degree to everyone, whether "sane," "insane," or "mentally defective."

In the case of a large number of these defective criminals, it is clear that they have been defective from their earliest years, and in cases where this fact cannot be established for certain, little difficulty presents itself in deciding whether or no the criminal's conduct gives evidence of mental defect apart from the commission of the crime for which he is sentenced. Without enlarging further on this important matter, I would call attention to the fact that, since the passing of the Preventive Detention Act a few years ago, which provided for the detention of criminals proved to be "habitual" for periods not exceeding ten years, several criminals who had been in previous sentences classed and treated as "mental defectives" in a special department of the convict prison at Parkhurst have been further sentenced, for fresh crimes, to penal servitude and to subsequent "preventive detention." Their previous treatment *in prison* as "mental defectives" was, of course, rendered nugatory by their ultimate release on expiry of their penal sentence. This patent evil will in all probability be remedied efficiently by the provisions of the Mental Deficiency Act as it stands.

### II. *The Act in its Relation to the Treatment of Mentally Deficient Children.*

It was made clear by the Report of the Royal Commission on the Care and Control of the Feeble-Minded (1908) that in spite of the existing "Lunacy" and "Idiots" Acts, and of the more recent Act concerning "Defective Children," by which "special schools" under the Board of Education were permissively established in several places by local authorities, large numbers of demonstrably defective children were left neglected and uncontrolled; and, further, that considerable numbers of hopelessly defective children were treated in special schools with inadequate results and at a cost which was largely unremunerative. Although there is no provision in the new Act for directly placing children who are found by expert medical evidence to be unfit for school life under the care of the Board of Control for Mental Defectives, it may be hoped that the Act as it stands will provide some facilities towards this end or may be further amended in this direction.

It is certain that the existing neglect and want of control of large numbers of mentally defective children and young persons leads to the commission of much preventable crime; to multiform misery; to the ruin of many girls; and to the birth of numbers of illegitimate children, among whom, according to good evidence, there is an undue proportion of the mentally deficient. These things being so, it is certain that this Act, in so far as it may help to ensure the careful custody of neglected children who are the subjects of permanent mental defect, will be of great practical value, and it may be hoped that if it prove inefficient in this direction the Act will soon be amended. Being abroad as I write this and unable to refer to the new Act, my memory of its provisions under this head may be incorrect. My impression, however, is that arrangements for the efficient care and control of defective children and young persons who are neither absolutely destitute, nor have come into conflict with the law, are inadequate for the prevention of much harm both done and suffered by the mentally deficient.

III. *The Working of the Act in Relation to the Study and possible Limitation of the Numbers of Persons who are the Subjects of Mental Defect.*

This subject must be mentioned shortly owing to the directly opposite views which have been expressed on the advisability or otherwise of establishing restrictions on the procreation of children by mental defectives, other than such incidental disabilities which must follow on the detention of such persons for their own safety and that of the community at large.

In the discussions which have taken place about the Mental Deficiency Bill all kinds of views have been put forward in this context. Extremists on the one hand have declared that however effective legislation might be in stamping out mental defect, the "liberty" of the mentally defective "subject" should take precedence of any general advantage to the community; while, on the other hand, some extremist supporters of "eugenics" have boldly advocated stringent legislation in the interest of the community on the gratuitous assumption that science justifies the conclusion that by such means "mental defect" could be permanently abolished.

The Act contains no provision explicitly directed towards this end, and in my opinion the omission of such a provision is right, at least in the present state of scientific knowledge of the subject. Although there is a vast amount of evidence to show that markedly deficient persons contribute far more largely to the mass of births of deficient children than the average of the population; and although there is good reason to believe that the great majority of births of deficient children are due to innate or germinal changes, yet, as a matter of fact, many births of mental defectives occur in families of all sorts and conditions in which no previous case can be traced in any of the near relatives. Such scientific knowledge of the genesis of marked mental defect as can with justice be practically applied to direct legislative measures is, in my judgment, not forthcoming in a degree sufficient to justify such crude and ill-conceived enactments which have been passed, even if they have not been widely employed, in some of the American States. Further knowledge may possibly lead to justifiable action in this direction in certain cases; but there is no indication, in the present state of science, that marked mental defect, however great its tendency to be reproduced in offspring may be, can ever be stamped out or prevented from occurring, as it were, *de novo.*

Nevertheless the due control of existing mental defectives, who would be otherwise neglected and at large and harmful to themselves and others, must affect to a considerable degree the total number of defective births.

IV. *The above considerations lead me to make a few remarks, in conclusion, concerning the possibilities provided or rendered possible by this new Act, of encouraging further scientific study of the subjects of mental defect themselves, as well as of their family histories.*

As much of the work of certifying and providing for mental defectives under the new Act will fall upon local authorities, it would appear highly desirable, not only for the efficient carrying out of the Act, but also in the interest of further knowledge, that specially chosen medical officers should be appointed to assist every local authority which deals with mental defectives. A recommendation to this effect was made by the Royal Commission, but, probably owing to reasons of finance, was omitted from the Act.

Further, it seems in the highest degree desirable that one at least of the members of the new Board of Control for Mental Defectives should be a scientific medical man thoroughly conversant with the present state of physiological and psychological knowledge of the subject, and thus able to stimulate and direct such studies at certain of the institutions for mental defectives as would be calculated to add greatly to existing knowledge and to give increased precision to practice. A valuable representation to this effect was recently made in the *Times* by Mr. Brudenell Carter and supported by a large number of distinguished physicians and surgeons.

There can be no doubt that this Act, as it stands, is one of the most useful and important legislative measures which have been passed in recent times.

